# AP endonuclease 1 (Apex1) influences brain development linking oxidative stress and DNA repair

**DOI:** 10.1038/s41419-019-1578-1

**Published:** 2019-04-25

**Authors:** De-Sheng Pei, Pan-Pan Jia, Juan-Juan Luo, Wei Liu, Phyllis R. Strauss

**Affiliations:** 1 College of Life Science, Henan Normal University, Xinxiang, 453007 China; 2 Key Laboratory of Reservoir Aquatic Environment, Chongqing Institute of Green and Intelligent Technology, Chinese Academy of Sciences, Chongqing, 400714 China; 3 Department of Biology, College of Science, Northeastern University, Boston, MA 02115 USA

**Keywords:** Enzyme mechanisms, Apoptosis

## Abstract

Brain and neurons are particularly sensitive to reactive oxygen species (ROS). Oxidative damage from ROS results in increased 8-oxoguanine in DNA followed by repair through the base excision repair (BER) pathway. We reported earlier that AP endonuclease 1 (Apex1) not only participates directly in BER but also regulates transcription factor Creb1. Here, we investigated how Apex1 affects brain to respond effectively to oxidative damage during zebrafish development. Loss of Apex1 resulted in increased ROS, 8-oxoguanine, and abasic sites as well as loss of Ogg1, which recognizes 8-oxoguanine and is required for its repair. Moreover, knock-down of Apex1 not only resulted in reduction of expression of several major proteins in the BER pathway (Polb and Ogg1), and it also resulted in maldistribution and loss of four key brain transcription factors (*fezf2*, *otx2*, *egr2a*, and *pax2a*), leading to abnormal brain development. These results were independent of p53 protein level. In contrast, exposure to exogenous H_2_O_2_ resulted in *increased* transcription and protein of Apex1 along with other BER components, as well as Creb1. Taken together, these results indicate that oxidative stress increased when the level of Apex1 was reduced, revealing a novel pathway of how Apex1 manages oxidative stress in developing brain.

## Introduction

The brain is exquisitely sensitive to oxidative stress^1^, never more so than in the developing embryo^2,3^. Oxidative stress can arise internally, since all cells produce oxygen free radicals in the form of reactive oxygen species (ROS) as byproducts of ATP synthesis during oxidative phosphorylation. Oxidative stress also originates from external sources, such as nitric oxide or peroxide. ROS damage protein, lipid, carbohydrate, and RNA, all of which can be resynthesized. However, unless oxidatively damaged DNA is repaired, genetic information could be lost, cells might die, or they might become transformed. Although large amounts of ROS are deleterious, small amounts are particularly important during early embryogenesis^4^ and are required for microglial activation and self-renewal^5,6^. Therefore, generation of ROS and repair of oxidatively damaged DNA are tightly regulated processes.

Most DNA damage arising from oxidative damage is repaired by the base excision repair (BER) pathway^7,8^. BER also repairs small lesions arising from alkylation, deamination, depurination/depyrimidination, and the presence of uracil^9,10^. Several proteins that participate in the BER pathway are embryonic or peri-embryonic lethals in mice, most notably AP endonuclease 1 (Apex1), DNA polymerase ß (Polb), XRCC1, and flap endonuclease 1 (Fen1)^11–14^. Furthermore, uracil DNA glycosylase (Ung), which recognizes uracil and serves as an entry point to BER, is an embryonic lethal in zebrafish^15^. Zebrafish embryos in which Apex1 has been knocked down completely arrest at the midblastula transition (MBT), when zygotic transcription is initiated and differentiation begins. Embryos in which the Apex1 or Ogg1 protein has been partially knocked down (hypomorphs) show defective brain and heart development^16,17^.

Recent evidence from this laboratory^18^ indicates that Apex1 is responsible for maintaining transcript and protein levels of the transcription factor Creb1 (cAMP response element-binding 1) and its binding partners Crem (Creb modulator), Torc1 and 3 (Creb regulator transcription co-activator 1 and 3), and CBP (Creb binding protein). Furthermore, damage to the Creb consensus sequence that is repaired by BER can affect Creb1 binding both positively and negatively^19,20^. Creb1 has a close relationship with normal brain development and neuronal function^21–25^, while CBP is known to regulate differentiation and survival of interneurons^26^. Apex1 regulates levels of DNA polymerase ß protein (Polb), the next participant in the BER pathway^18^, *via* Creb1. Indeed, Creb1 activity is also associated with modulating neural cell proliferation, midbrain–hindbrain organization, and patterning^27^.

Apex1 is an excellent marker for rapid proliferation in cancer cells including glioma, prostate, head and neck, pancreas, colon and breast^28–33^. Consequently, it has frequently been marked as a potential target for chemotherapy^34^. Not surprisingly, most, if not all, of the transcription factors with which Apex1 is known to interact by various methods, including AP-1^35^, Jag1^32^, Egr1^32^, Mdm2^36^, p53^37^, HIF-1^38^ and NF-kB^39^ among others, are directly or indirectly dependent on Creb1 for regulation of expression and their upregulation has been associated with poor outcomes for cancer chemotherapy. Creb1 plays a vital role in the central nervous system, and genetic disruption of Creb1 leads to neurodegeneration in brain^40^. Recently, brain-derived neurotrophic factor (BDNF) was reported to activate Creb1 and upregulate Apex1 in the cerebral cortex and hippocampus of mice^41^. However, to date, there have been no documented reports about how apex1 regulates the brain development.

In this study we demonstrate that loss of Apex1 results in increased generation of ROS and decreased *creb1* expression, leading to aberrant brain development. Since the changes are independent of p53, they do not fit the profile of p53-mediated off-target effects and argue for Apex-related regulation of Creb1. We propose that independently of p53, Apex1 enables brain and neurons to respond effectively to oxidative damage and minimize cancer progression, thereby serving as a master regulator of brain development through its control of Creb1.

## Results

### Knocking down Apex1 protein results in increased oxidative stress and oxidative damage to DNA

**Fig. 1:**
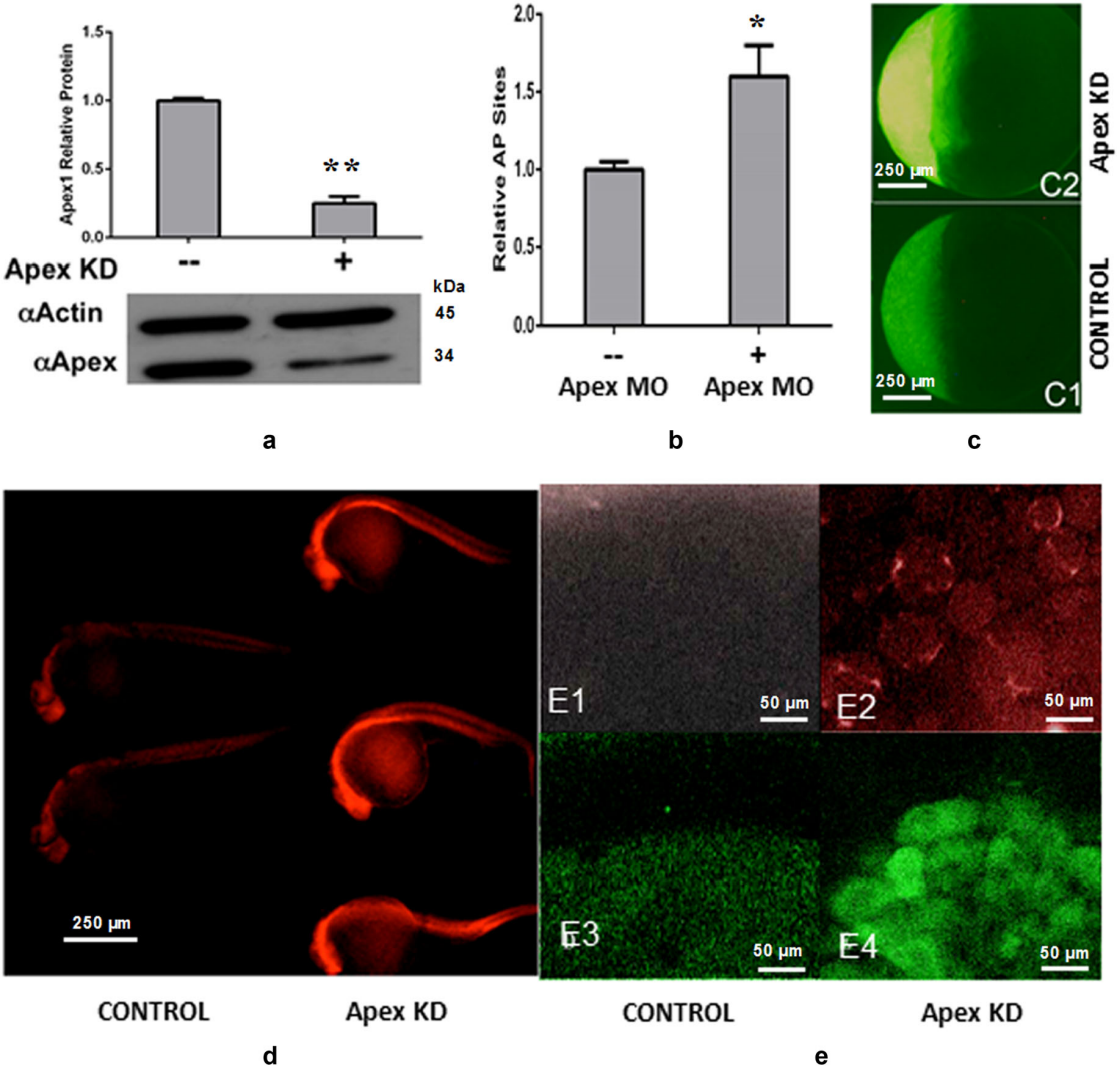
Loss of Apex1 protein results in increased oxidative damage, AP sites and ROS. **a** Western blot analysis of Apex1 knockdown by morpholino (MO). Upper panel, quantitative analysis of WB. Significant difference is indicated by **p *<* 0.01. **b** Increased apurinic/apyrimidinic (AP) sites relative to those in controls after Apex1 knockdown. Data represent the average of five independent experiments ± SD of the mean. Significant difference is indicated by *p *<* 0.05 and **p < 0.01. **c** Increased ROS after Apex1 knockdown detected by CM-H2DCFDA. Embryos were microinjected with control MO (panels C1) or 0.2 mM Apex1 MO (panels C2), exposed to CM-H2DCFDA to detect generalized ROS, washed and examined by fluorescence microscopy. Fluorescence was greatly increased in Apex1 knockdown embryos. Photographed at ×4 magnification. **d** Increased oxidative DNA damage in 24 hpf embryos after Apex1 knockdown. °G levels, detected by immunostaining with TRITC-labeled anti °G mouse monoclonal antibody. Apex1 knockdown embryos (right three embryos) and control embryos (left two embryos) were examined by fluorescence microscopy. These experiments were repeated three times with similar results. **e** Detection of superoxide anion using MitoSOX red (panels **e1** and **e2**) and nitric oxide using DAF-FM acetate (panels **e3** and **e4**). Embryos were microinjected with vehicle (panels **e1** and **e3**) or Apex1 MO (panels **e2** and **e4**), examined at 4 hpf by confocal microscopy and photographed at ×40 magnification. Note perinuclear mitochondria containing superoxide in Apex1 knockdown embryos (**e2**) and the appearance of nitric oxide in the yolk syncytial layer in occasional Apex1 knockdown embryos (**e4**)

Oxidative damage to DNA, whether from endogenous or exogenous sources, generally requires repair by the BER pathway in order to maintain genome integrity^42,43^. Since loss of Apex1 also results in loss of Polb, the next protein in the BER pathway, due to loss of Creb1^18^, we examined whether Apex1 loss resulted in accumulation of oxidative damage to DNA in early zebrafish embryos. Two sensitive parameters for oxidative damage to DNA are increased levels of 8-oxoguanine (°G)^44^, and abasic (AP) sites in DNA. Apex1 MO microinjected within three doublings after fertilization (2 h post fertilization, hpf) dramatically decreased the Apex1 protein level detected at 24 hpf (Fig. 1a) and increased AP sites detected in extracted DNA as measured by aldehyde reactive probe (Fig. 1b). It also resulted in increased presence of °G (Fig. 1d). Thus, loss of Apex1 correlated well with increased oxidative damage to DNA.

To explore the source of oxidative damage in embryos that were hypomorphic for Apex1, we asked whether we could detect endogenous ROS at 6–7 hpf by means of several fluorescent dyes that are activated by specific ROS. Three vital dyes were used: generic ROS were detected using CM-H2DCFDA; superoxide anion was detected by means of Mitosox Red, and nitric oxide was detected using DAF-FM acetate. Oxidative stress levels were markedly enhanced in Apex1 knockdown embryos. Major amounts of ROS accumulated in Apex1 MO knockdown embryos, compared to controls (Fig. 1c). While some superoxide, detected by Mitosox Red, appeared in perinuclear mitochondria (Fig. 1e, compare panels **e1** and **e2**), occasional knockdown embryos showed the presence of NO in the syncytial yolk layer (Fig. 1e, compare panels **e3** and **e4**). These results taken together indicated that oxidative stress increased, when the level of Apex1 was reduced.

### Loss of Apex1 is associated with ventricle deformity and mal-distribution of key brain transcription factors

**Fig. 2:**
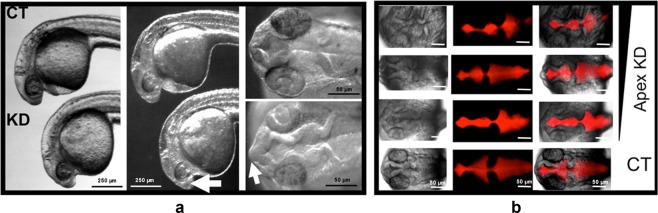
Loss of Apex1 protein in WT embryos alters brain morphology. **a** Brain abnormalities at 24 hpf after Apex1 knockdown. Embryos microinjected with Apex1 MO (KD) had enlarged forebrains, indicated with arrows. Darkfield imaging (middle panels) highlights forebrain abnormality (arrow) in KD compared with control embryos. Dorsal views of KD embryos (third panel) show abnormality of the brain ventricle in knockdown embryos relative to controls. **b** Abnormal ventricle space shown by Texas Red dextran depends on the concentration of Apex1 MO (0.1, 0.15, or 0.2 mM) used for knockdown. Dye was microinjected directly into the ventricle space at 24 hpf. These experiments were performed three times with similar results

Since Apex1 regulates transcription and protein levels of Creb1 and its binding partners^18^, and since Creb1 is critical for brain and neuronal development^25,27^, we explored the role of Apex1 in zebrafish brain development in greater detail. The zebrafish embryonic brain begins as a simple tube, the lumen of which forms the brain ventricle and fills with cerebrospinal fluid^45^. In addition to having smaller heads and eyes, embryos microinjected with Apex1 MO had enlarged forebrains, compared with controls (Fig. 2a). Abnormality of the brain ventricle was further revealed by injecting Texas Red conjugated to dextran directly into the ventricle at 24 hpf^46^. The severity of the deformed ventricle space was readily observed in Apex1 knockdown embryos and depended on the degree of Apex1 knockdown (Fig. 2b).

**Fig. 3:**
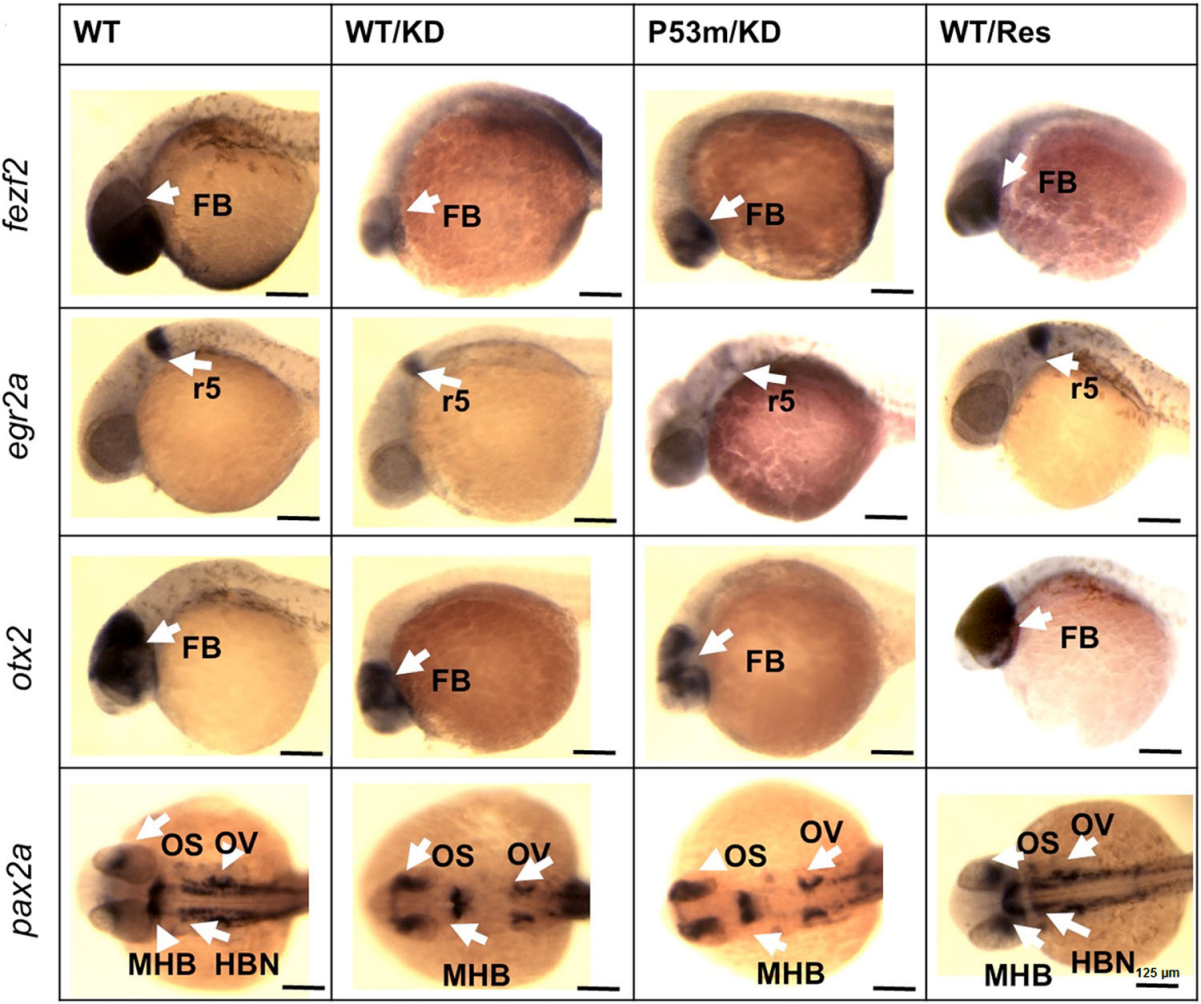
Whole mount in situ hybridization demonstrates reduction in four key brain transcription factors after Apex1 knockdown in both wild-type and p53 mutant embryos with rescue by co-injection of mRNA for human *APEX1*. Whole mount in situ hybridization shows aberrant distribution of critical brain markers after Apex1 knockdown. Rescue was achieved by co-injection of transcript for human *APEX1*. Similar results were obtained in p53 mutant embryos in which Apex1 was knocked down. Whole mount in situ hybridization was performed to examine *fezf2*, *otx2*, *egr2a*, and *pax2a* expression after knockdown of Apex1 in wild-type and p53 mutant embryos. Expression of each transcription factor decreased, and distribution was altered in both Apex1 MO injected wild-type and p53 mutant embryos, but was rescued by co-injection with human *APEX1* capped mRNA. Note the small heads and eyes in Apex1 knockdown embryos. Hindbrain neurons (HBN) indicated by *pax2a* expression were no longer visible in Apex1 MO injected embryos (*pax2a* panel). Alteration in distribution or amount of signals is marked with arrows or brackets. KD knock down, WT wild-type, Res Apex1MO + human Apex1 rescue, p53m p53 mutant embryos, FB forebrain, MB midbrain, r5 hindbrain rhombomere 5, OS optic stalk, MHB midbrain-hindbrain boundary, OV otic vesicle. Whole mount in situ hybridization was performed with 20 embryos/group. All embryos are shown with anterior to the left

In addition, we performed in situ hybridization to examine the distribution of the individual genes in WT and p53 mutant embryos after knockdown of Apex1. These genes, which are critical for neural patterning and contain Creb1 sites in their promoters (Supplementary Fig S1), include forebrain (FB) (*fezf2*, FEZ family zinc finger 2), midbrain (MB) (*otx2*, orthodenticle homolog 2), MB-hindbrain (HB) boundary (*pax2a*, paired box gene 2a), and HB rhombomere 5 (*egr2a*, early growth response 2a). Zebrafish paired box (*pax*) genes are particularly relevant, because they encode transcription factors that regulate differentiation of cells in the central nervous system (CNS) and are expressed in the early neural tube^47^. Not only was mRNA of each of the four genes reduced after Apex1 knockdown (Supplementary Fig S2), but also mRNA of all four transcription factors in knockdown embryos was abnormally expressed (Fig. 3). Of particular note, hindbrain (HB) neurons, marked with antisense *pax2a* probe, could not be visualized in the Apex1 MO injected embryos. Expression level of rhombomere 5 (*r5*) stained by *egr2a* probe dramatically decreased in Apex1 knockdown groups of both wild-type and p53 mutant embryos. Forebrain markers of *fezf2* and *otx2* were greatly reduced after loss of Apex1. Co-injection of capped human *APEX1* mRNA along with MO directed against zebrafish Apex1 rescued the defects. Similar aberrations were observed in p53 mutant embryos (p53m) (See below).

### Loss of Apex1 decreases the expression profiles of Polb and Creb1 in zebrafish brain

**Fig. 4:**
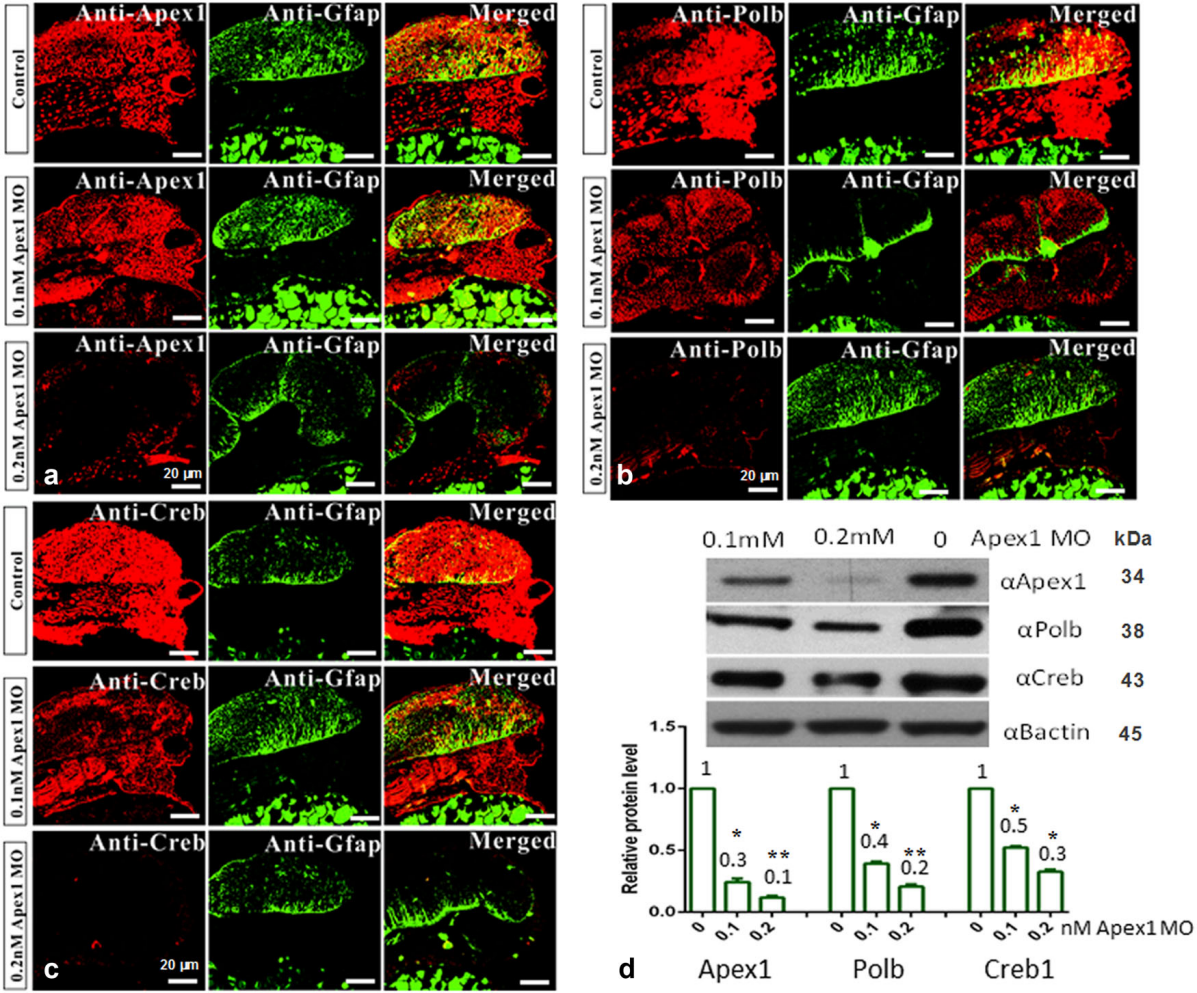
Fluorescent immunohistochemical staining of zebrafish brain for Apex1, Polb, and Creb1. Standard fluorescent immunohistochemical staining was performed, and recorded by confocal microscopy (×40 magnification). Zebrafish embryos at the 1-cell stage were injected with control MO, 0.1, or 0.2 mM Apex1 MO, and allowed to develop for an additional 48 h before they were fixed and processed as described in Methods. Green fluorescence represents the expression pattern for glial fibrillary acidic protein (GFAP), while red fluorescence indicates the expression pattern for the indicated protein: Apex1 (**a**), Polb (**b**), or Creb1 (**c**), respectively (*n* = 20 in each group). **d** Western blot quantification of Apex1, Polb, or Creb1 (*n* = 30). Each experiment was repeated three times. Significant difference is indicated by *p *<* 0.05 and **p < 0.01

To reveal the relationships between the BER pathway and brain development, immunofluorescence was assayed using double-staining with GFAP (glial fibrillary acidic protein, a popular marker of astroglia in the brain) and major enzymes of BER (Apex1 or Polb). Creb1, previously reported to participate in the BER pathway^7^, was also examined. As shown in Fig. 4, injection of 0.2 mM Apex1 MO dramatically decreased Apex1 expression in zebrafish brain (Fig. 4a), and also critically reduced expression levels of Polb and Creb1 (Fig. 4b, c), which was further confirmed by western blot assay (Fig. 4d). Moreover, results showed that loss of Apex1 affected the expression profiles of Polb, and Creb1 in an Apex1 MO concentration-dependent manner. GFAP was specifically expressed in the central nervous system (CNS) in control and knockdown astrocyte cells. While Apex1, Polb, and Creb1 were expressed in all cells, which reflects their roles in maintaining basic cellular function (Fig. 4a–d), they were markedly depleted in brains of Apex1 knock down fish.

### Loss of Apex1 affecting zebrafish brain development is independent p53

**Fig. 5:**
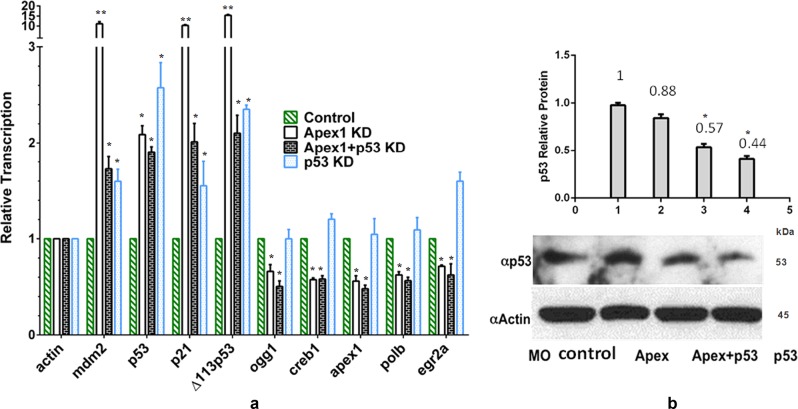
Effects on transcription of selected marker genes after knockdown of Apex1 do not correlate with changes in p53 transcription. **a** Alterations in transcription after knockdown of Apex1, p53 or Apex1 and p53 together shown by qRT-PCR at 24 hpf. Embryos were microinjected at the 1–2 cell stage with the indicated MO or vehicle (control) and harvested at 24 hpf for RNA extraction. ß-actin was used as the endogenous reference. Creb1-dependent gene expression including *ogg1, creb1, polb and egr2a*, was diminished at 24 hpf whether or not the level of p53 mRNA had increased and despite the loss of p53 protein in p53 knockdowns and p53 mutant fish (panel **d**). Error bars represent standard deviation from the mean for 3 experiments. Significant difference is indicated by *p *<* 0.05 and **p < 0.01. **b** Western blot analysis of protein extracts from wild-type embryos after Apex1 knockdown, Apex1+p53 knockdown, or p53 knockdown alone. Upper panel: quantitative analysis. Although p53 protein remained relatively unchanged after Apex1 knockdown, it was significantly reduced from that of controls when either p53 alone or p53 and Apex1 together were knocked down. Data obtained from 50 embryos/group. Significant difference is indicated by *p *<* 0.05

To determine whether our results reflected a physiological function for Apex1 in brain development but not off-target effects of morpholino oligonucleotide injection, we performed qRT-PCR analysis to examine the expression levels of several apoptosis-related genes including *p53*, *p21*, *mdm2* and a dominant negative inhibitor of p53, *Δ113p53*^48^, in wild-type control embryos and in wild-type embryos after knockdown of Apex1, p53, or Apex1+p53 together (Fig. 5a). Simultaneously we examined the BER-related transcripts, *apex1, polb*, and *ogg1*, the latter two having been shown to be reduced by Apex1 knockdown in zebrafish^18^ as well as the critical brain development factors *egr2a* and *creb1*. In a separate set of experiments we compared these results to protein levels of p53 (Fig. 5b). Knockdown of p53 protein resulting from morpholino oligonucleotides targeting p53 resulted in >50% loss of p53 protein even as *p53*, *p21*, *mdm2*, and *Δ113p53* *transcripts* remained elevated. Knockdown of Apex1 alone only marginally altered protein levels of p53 but did result in greatly enhanced transcript levels of the three apoptosis-related genes. Knockdown of both Apex1 and p53 together resulted in nearly as much loss of p53 protein as knockdown of p53 alone. Even as p53 protein was diminished, transcript levels of p53-related proteins mirrored those after *apex1* knockdown alone. Note that transcripts of the dominant negative *Δ113p53* were markedly upregulated whenever embryos were subjected to Apex1 or p53 knockdown. In contrast to effects on the four genes known to be apoptosis-related, transcript levels of *creb1*, *polb*, and *ogg1* mRNA levels were reduced relative to controls (Fig. 5a). In fact, simultaneous knockdown of Apex1 and p53 resulted in hypomorphs with apoptosis-related gene transcripts returned nearly to control levels, while *creb1*, *polb* and *ogg1* levels had not recovered (Fig. 5a). To reiterate, despite the fact that levels of *p53* mRNA were still somewhat increased even in p53 knockdown embryos, p53 protein levels were markedly reduced in p53 and p53+Apex1 knockdown embryos (Fig. 5b).

To further explore the apparent inverse relationship between canonical p53-related gene expression and Apex1-mediated effects on brain development, we performed similar Apex1 knockdown experiments in wild-type and p53^M214K/ M214K^ embryos. These embryos fail to respond to ionizing radiation exposure with enhanced apoptosis^49–51^ and have been used to avoid off-target effects of MOs^49,50^. These embryos manifested the same morphological changes upon Apex1 knockdown as wild-type embryos (Fig. 3 and Supplementary Fig S3). We again analyzed the degree of expression of the four well-characterized genes noted above and that are heavily involved in neural patterning. The distributions and amounts were again abnormal and mirrored the changes observed in single Apex1 knockdown embryos. As before, rescue of all four was achieved by co-injection of human *APEX1* mRNA along with MO directed against zebrafish Apex1 (Fig. 3 and Supplementary Fig S3). Since the embryonic abnormalities arising after Apex1 loss were largely independent of p53-mediated processes, occurring both in double knockdowns of p53 wild-type embryos and in p53-mutant embryos and also with rescue by co-injection of capped mRNA encoding human *APEX1*, they were not the result of p53-mediated events and cannot be considered an off-target effect.

### Creb1 is a major transducer of Apex1 in regulating brain development responding to oxidative damage

**Fig. 6:**
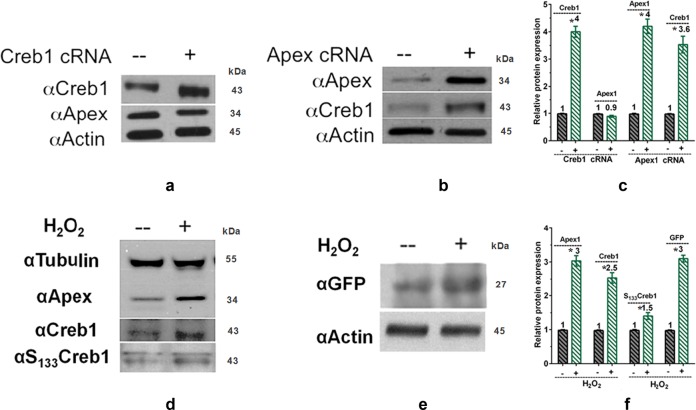
Overexpression of Apex1 upregulates Creb1 but not vice versa; exposure to exogenous ROS from H_2_O_2_ upregulates both Apex1 and Creb1. **a**, **b** Western blot analysis demonstrated that overexpression of Creb1 (microinjection of *creb1* capped mRNA) did not affect Apex1 protein levels (**a**), but overexpression of *apex1* capped mRNA (microinjection of *apex1* capped mRNA) increased Creb1 protein levels. *apex1* or *creb1* capped RNA (540 pg) was microinjected into 1–2 cell stage embryos, and protein extracts were prepared at 24 hpf. Representative blots are shown. **c** Quantification of data from three independent experiments. Error bars are the standard error of the means (SEM). Significant difference is indicated by *p *<* 0.05. **d** Exposure to 1.5% hydrogen peroxide for 0.5 h results in increased protein levels of Apex1, total Creb1, and Ser^111^ phosphorylated Creb1 in zebrafish embryos at 6 hpf. **e** Hydrogen peroxide activates Apex1 expression *via* the Apex1 promoter. The constructed pApex1-eGFP plasmid was microinjected into 1–2 cell embryos (0.1 ng/embryo), which were then exposed to fish water or fish water containing 1.5% H_2_O_2_ for 0.5 h. Extracted protein at 24 hpf was examined by western blot analysis for GFP and ß-actin. Western blot analysis showed increased GFP in the H_2_O_2_ treated group. **f** Quantification of western blot results shown in (**d**) and (**e**). Significant difference is indicated by *p *<* 0.05

Genetic and pharmacological studies in mice and rats demonstrate that Creb1 is a universal modulator of processes required for memory formation^22^, neural cell proliferation, MB-HB organization, and patterning in zebrafish^27^. To confirm the relationship between Creb1 and Apex1 in regulating brain development, western blot analysis was used to examine alterations in Creb1 and Apex1 proteins after injection of capped mRNA encoding each gene. The result showed that overexpression of *creb1* did not affect Apex1 protein levels, but overexpression of *apex1* increased Creb1 protein levels, implying that Creb1 is likely to be a major transducer for Apex1 in responding to oxidative damage and in regulating brain development (Fig. 6a, b). Furthermore, overexpression of Creb1 substantially restored 24 hpf viability of embryos lost upon Apex1 knockdown (Supplementary Fig S4).

### Exposure to exogenous ROS activates the BER pathway

If loss of Apex1 results in endogenous generation of ROS with resulting brain and heart abnormalities, how might the developing embryo respond to exogenous ROS? To elucidate how Apex1 responds to oxidative damage from exogenous sources, embryos were exposed to a common environmental oxidant, H_2_O_2_, and processed either by immunostaining with TRITC-labeled anti °G mouse monoclonal antibody to document oxidative DNA damage or for western blot analysis to analyze the key protein levels involved in the BER pathway. Treatment with 1.5% H_2_O_2_ for 30 min at 6 hpf (shield stage) resulted in small eyes and a flattened head at 24 hpf similar to that seen in Apex1 hypomorphs (Supplementary Fig S5). Along with the changes in morphology, this treatment resulted in increased protein levels of Apex1, total Creb1 and Ser^111^ phosphorylated Creb1 at 24 hpf (Fig. 6d, f). To explore this result further, the 3 kb *apex1* promoter was cloned from zebrafish genomic DNA into the pApex1-eGFP plasmid in order to analyze *apex1* promoter responses to oxidative damage arising from exogenous sources. Western blot analysis showed that the GFP protein level driven by the *apex1* promoter increased in the H_2_O_2_ treated group (Fig. 6e, f). Thus, Apex1 promoted a strong response to exogenous oxidant but was insufficient to offset the damage arising under these conditions.

**Fig. 7:**
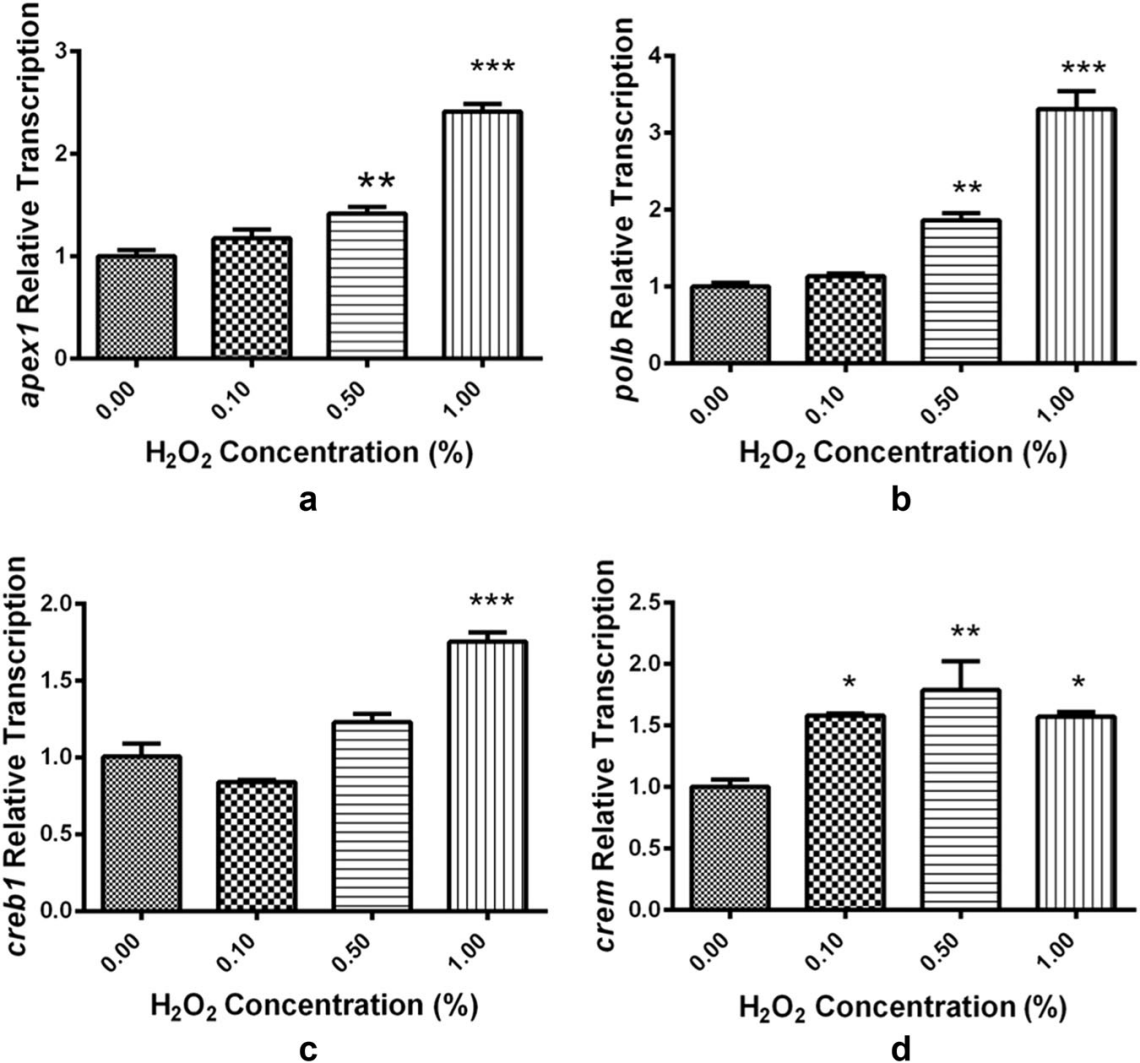
Exposure to hydrogen peroxide (H_2_O_2_) activates base excision repair (BER) pathway in zebrafish embryos. **a**–**d** Transcript levels of four critical BER genes (*apex1*, *polb*, *creb1*, and *crem*) quantified by real-time PCR immediately after exposure to the indicated concentration of peroxide. Transcript levels of *apex1*, *polb* and *crem* were significantly increased after exposure 0.5, and 1% H_2_O_2_. The results are means ± SEM of three replicate samples. Significant difference between exposure groups and the control groups is indicated by *p < 0.05 and **p < 0.01 and ***p < 0.001

To further investigate how exogenous ROS activates the BER pathway, we exposed 2 hpf embryos for 4 h to a range of concentrations of peroxide in order to examine the effects of overwhelming the embryos with exogenous ROS. As noted earlier, qRT-PCR analysis showed that MO-mediated knockdown of Apex1 decreased *ogg1* and *polb* transcription levels at 24 hpf. In contrast, after exposure to 0, 0.1, 0.5, and 1% H_2_O_2_, the transcript levels of *apex1* and three genes dependent on Apex1, namely *polb*, *creb1*, and *crem* increased with increasing H_2_O_2_ concentration (Fig. 7a–d**)**. In short, Apex1 regulated the embryo’s response to oxidative stress at multiple levels.

## Discussion

In this study, we have shown that Apex1 regulates the balance between oxidative stress and the response to oxidative damage in developing zebrafish. Regulation occurred through control of transcription of Creb1. Loss of Apex1 resulted in enhanced levels of reactive oxygen species and increased °G in DNA, a marker for oxidative DNA damage, despite the fact that WT zebrafish Apex1 lacks redox activity as defined by Kelly et al.^52^. Not only were ROS levels increased in DNA after Apex1 loss, but also transcripts of Ogg1, the enzyme that recognizes oxidative DNA damage and initiates BER, were diminished in a Creb1-dependent fashion. Brain abnormalities resulting after reduced Apex1 levels included small heads, distortions in the ventricle, and reduced expression and maldistribution of several typical brain markers (*fezf2, otx2, egr2a*, and *pax2a*). While overexpression of *apex1* restored *creb1* levels, overexpression of *creb1* only marginally restored depressed *apex1* levels. These effects were independent of p53, since they occurred in wild-type, p53 mutant and double MO knockdown (p53 along with Apex1) embryos. In contrast to the effects stemming from Apex1 loss, exposure of early embryos to exogenous ROS in the form of H_2_O_2_ resulted in increased transcript levels of *apex1, polb, creb1*, and *crem* levels as well as increased protein levels of Apex1 and Creb1.

In earlier work we reported that loss of Apex1 results in failure of Polb, the next enzyme in the BER pathway, to appear at the expected time in development. The failure occurs *via* a Creb1 dependent mechanism^18^ in both wild-type and p53 mutant embryos. Rescue requires the coding sequence for the endonuclease competent enzyme. These results are widely applicable, since Creb1 levels are diminished in primary cultures of B cells from *apex1*^+/−^ mice when exposed to inhibitors of residual Apex1^18^. Taken together with the results provided in this report, our data indicate that Apex1 controls the major proteins in the BER pathway in vertebrates by a mechanism requiring cleavage of an abasic site in DNA. Many transcription factors involved in brain development, including the ones studied here, have Creb1 binding sites in their promoters. Since the presence of unrepaired AP sites in the Creb consensus sequence is highly deleterious to Creb binding^19,20^, repair of oxidative damage to DNA is inexorably linked to Creb1-dependent processes in brain development.

In most cultured cells, reduction in Apex1 levels results in upregulation of p53 and initiation of apoptosis, although a few cultured cells seem refractory to the apoptotic response^37,53^. Consistent with these studies, some tissues in developing zebrafish embryos like brain and heart are far more sensitive to Apex1 knockdown than others such as muscle^16^. Nevertheless, the use of morpholino oligonucleotides in zebrafish for knocking down expression of selected genes can be complicated by off-target effects leading to apoptosis. To overcome these obstacles, we performed experiments in p53 mutant fish^51^ and in wild-type fish with simultaneous knockdown of p53 along with Apex1^50^. We also rescued the knockdown with the messenger RNA encoding Apex1. We report here that the Apex1 knockdown phenotype arises not only in p53 mutant fish but also in double knockdowns of p53 and Apex1 in wild-type embryos and is rescued by co-injection of the mRNA for Apex1. Therefore, off-target effects due to use of morpholino oligonucleotides are unlikely to be responsible for the Apex1 knockdown phenotype. Apex1 (also known as Ref1) is well known in cultured cells to stabilize p53 binding to its cognate sequence^54^. Decreased binding efficiency due to loss of Apex1 could shift the target specificity of this complex protein, diminish the apoptotic response and/or enhance the ability of the dominant negative *Δ*113p53 to compensate for loss of p53 protein.

### Apex1 mediates brain development

**Fig. 8:**
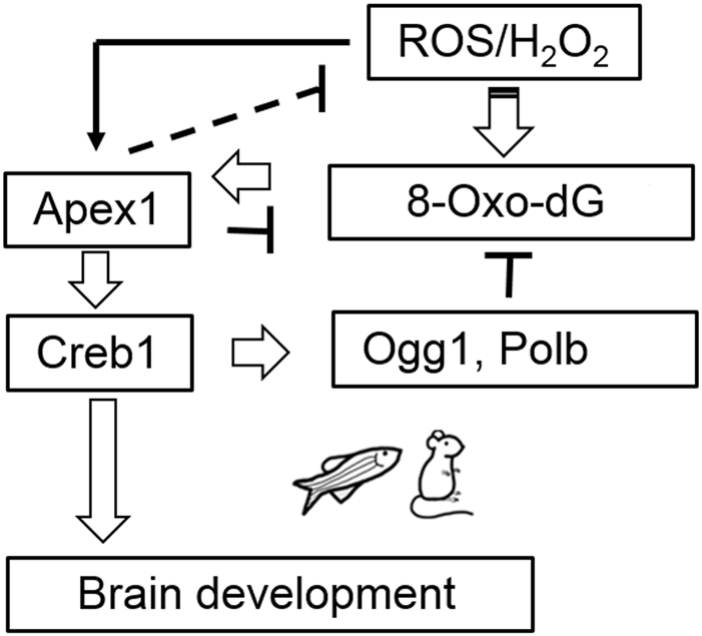
Schematic diagram of how Apex1 responds to oxidative damage to regulate brain development *via* Creb1. ROS result in oxidative damage to DNA, including increases in °G and AP sites, which enhance protein levels of Apex1. Increased Apex1 results in increased Creb1 that in turn enhances levels of BER pathway participants (Ogg1 and Polb), which together with Apex1 repair oxidatively damaged DNA. Meanwhile, other genes dependent on Creb1 are also upregulated, many involved in development, particularly brain development and function, including *pax2*, *egr2a*, *fezf2*, and *otx2*

Given the importance of appropriate balance between oxygen levels and antioxidant status in both fish and mammals^4^, our data lead us to formulate the hypothesis shown in Fig. 8. Apex1 regulates protein levels of Creb1 and its binding partners; Creb1 regulates ~5000 genes, including those encoding Polb and Ogg1, and ~60 transcription factors. Loss of Apex1 leads to increases in endogenous ROS, and °G levels along with AP sites in DNA. Therefore, Apex1 regulates the entire BER pathway by controlling the protein levels of many participating repair enzymes.

Exposure of embryos to exogenous ROS increases *apex1*, and *creb1* expression, comprising a feed forward loop designed to protect the organism against harm caused by excessive oxidation. As ROS are inevitable byproducts of oxidative phosphorylation, we propose that excessive levels of ROS are those that result in sufficient unrepaired DNA damage so as to overcome the feed forward loop and activate apoptosis^55,56^. Ogg1 is thought to protect neurons against oxidative DNA damage and cell death under ischemic conditions both in vivo and ex vivo (in culture)^57^. Although °G is a normal, though transient, component of oxidized DNA, it is not in and of itself toxic unless concentrations rise above a certain level^58^, as it only marginally interferes with transcription and Creb1 binding^19,20^. While it is unlikely that decreased levels of Ogg1 *per se* explain the effects seen after Apex1 knockdown, increased levels of °G might serve as a signal to augment the BER pathway.

Many aspects of brain development are conserved between zebrafish and mammals including various details of ventricle formation, as reviewed by Lowery^59^. In Apex1 hypomorphic embryos that survive hatching, the entire midbrain region is devoid of cells^16^, which is consistent with inadequate circulation of cerebral spinal fluid in addition to defects in expression of transcription factors required for normal brain development. Neurodevelopmental disorders associated with ventricle abnormalities include schizophrenia, autism, idiopathic and syndromal mental retardation, fragile X syndrome, Down’s syndrome, attention-deficit-hyperactivity disorder, and other learning disorders. Might these disorders involve diminished ability to repair oxidative damage?

### The importance of Apex1 as a mediator of Creb1-dependent pathways

There is no information on what regulates protein levels of Creb1, which itself controls ~25% of the eukaryotic genome. In this work we demonstrate that the DNA repair protein AP endonuclease 1 regulates Creb1 in response to oxidative DNA damage and thereby promotes normal development of vertebrate brain. As Creb1 dysregulation is likely to be involved in multiple neurological conditions and cancer metastasis, these results provide new insight into the importance of oxidative DNA damage and its repair. Although mammalian APE1 has been characterized as a redox factor in vitro, its zebrafish counterpart lacks the critical cysteine involved in redox activity^60^. While Ref1 (Ape1) null mice die very early in embryonic development^61^, as do Apex1 full knockdown zebrafish^16^, homozygous mice lacking the critical cysteine Cys^62^ required for the redox activity are viable, survive to normal life expectancy and display no overt abnormal phenotype. In addition, rescue of full Apex1 knockdown zebrafish requires the endonuclease competent message^16^ and homozygous redox deficient murine cells retain normal levels of endogenous AP-1 (Fos and Jun) DNA binding capacity. Thus, loss of the DNA repair activity involved in base excision repair is life threatening.

Apex1 has been linked to normal brain function in the past, though a mechanism remained unclear until now^63,64^. Creb1 and its binding partners play a well-documented critical role in nervous system development and in the protective response to neuron stressors^62^. Creb1 dysregulation is likely to be involved in Alzheimer’s disease^65^, Parkinson’s disease, Huntington’s disease, Rubinstein-Taybi syndrome, ischemia, alcoholism, schizophrenia, addiction, and depression (reviewed in Sakamoto et al.^62^ and Thakur et al.^66^). In the mature CNS, Creb1 mediates transcription of multiple factors required for neuronal survival. Although much effort has been invested in examining the role of various growth factors required for neuronal survival, the fact that Apex1 might itself control these same growth factors through Creb1 and link neuronal survival directly to repair of oxidative damage to DNA has not been appreciated.

In conclusion, we have demonstrated that Apex1 is central to the brain’s response to oxidative stress *via* its ability to participate in and regulate the base excision repair pathway. This regulation is independent of p53-mediated processes. These data are the first to link ROS to brain development and repair of DNA damage in a mechanistic fashion.

## Materials and methods

### Zebrafish husbandry, breeding, and knockdown of Apex1 and p53

Wild-type zebrafish were purchased from Aquatic Tropicals (Plant City, FL USA). Homozygous p53 mutant zebrafish adults (p53^M214K/M214K^) were kindly provided by Dr. Thomas Look^51^. In this line, fish fail to undergo DNA damage-dependent apoptosis after γ-irradiation, fail to upregulate p21 after UV irradiation and do not arrest at the G1/S checkpoint^51^. This line has been used to dissect off-target from targeted knockdown by morpholino oligonucleotides (MO)^49,50^. Wild-type zebrafish and homozygous p53 mutant zebrafish were maintained and bred as described^16,18,67^ in accordance with standards of the Division of Laboratory Animal Medicine of Northeastern University. Knockdown of Apex1 was performed by microinjecting 0.15–0.25 mM MO directed against the translation start site of *apex1* as described^16,18^. Control embryos were microinjected with the same vehicle used for microinjection of MO. Knockdown of p53 was performed by microinjecting 0.4 mM p53 MO (5ʹGCGCCATTGCTTTGCAAGAATTG3ʹ)^49^, since higher concentrations of the p53 MO were lethal when combined with 0.2 mM Apex1 MO. Because full loss of Apex1 protein is embryonic lethal at the midblastula transition^16^, both p53 and Apex1 knockdowns were hypomorphs. All experiments were performed two or three times with independent biological samples, as indicated.

### Western blot and quantitative real-time polymerase chain reaction (qRT-PCR)

Protein extraction, western blot, and qRT-PCR were performed as described^18^. Zebrafish specific anti-Apex1 antibody was prepared against zebrafish Apex1 residues 140–155 by Sigma-Genosys (The Woodlands, TX, USA)^16^. Polyclonal rabbit antibodies to quantify ß-actin, ß-tubulin, Creb1, p133Creb1, and GFP were obtained from Abcam, Inc. (Cambridge, MA, USA), while polyclonal rabbit antibody to quantify p53 was purchased from AnaSpec, Inc. (Fremont, CA, USA). Since results with ß-actin and ß-tubulin were identical, the choice of standard depended on being able to differentiate the standard from the protein to be quantified on gel electrophoresis. Data were analyzed by BandScan software. Wherever possible, standard and unknown were probed on the same blot with pooled antisera. We were unable to identify either a commercial or a custom antibody directed against Ogg1 that did not cross-react with other, unidentified zebrafish proteins. The primer sequences for real time PCR are listed in Table 1. For protein and RNA experiments, 75 and 50 embryos were harvested, respectively.

**Table 1:** Primers used in this study

Genes	GeneBank No.	Primer sequences	Size (bp)
*Δ113p53*	NM_131327	5-ATATCCTGGCGAACATTTGG-3	2214
		5-ACGTCCACCACCATTTGAAC-3	
*apex1*	NM_213421	5-AATAAAGTGTTGGGTGTACGTG-3	251
		5-CAGGAGGTGATCTTCATATTGG-3	
*Apex1* P	BX323558	5-CGACTCAGCGACCTTCTTGC-3	2956
		5-GTTTACAGTTGTTTTCAGGCCAC-3	
*β-actin*	NM_131031	5-CCCAGACATCAGGGAGTGAT-3	239
		5-TCTCTGTTGGCTTTGGGATT-3	
*creb1*	NM_200909	5-AGGAGCGTGGAGAACCATAAA-3	151
		5-GGCAGAGCCATCAGCGAC-3	
*egr2a*	NM_183341	5-GCCGTTTTCGTGCTCTTTG-3	127
		5-CGGGTTGGTGCCGTCTAA-3	
*fezf2*	NM_131636	5-TTTGTGGAAAGGTCTTCAACG-3	845
		5-CATGTTTGCTTTACTGCGTC-3	
*mdm2*	NM_131364	5-AGACTCTCGCTCATCTACCT-3	230
		5-ATATACCTACATCCGAGTTGCTG-3	
*ogg1*	NM_001123308	5-CAAGATCTTACAGACCCTTGTG-3	232
		5-CAAACTTGTCCAGTGACATCAG-3	
*otx2*	NM_131251	5-TTGGATACCCAGCGACTCCT-3	1116
		5-TCCGTGGCTTTGACCTAACTT-3	
*p21*	AL912410	5-CGGAATAAACGGTGTCGTCT-3	213
		5-CGCAAACAGACCAACATCAC-3	
*p53*	NM_131327	5-CTCTCCCACCAACATCCACT-3	178
		5-ACGTCCACCACCATTTGAAC-3	
*pax2a*	NM_131184	5-GGACACTGGAGCAGACGCA-3	1224
		5-CACGCTGGAGCCCAAATC-3	
*polb*	NM_001003879	5-TCCCTGAACGAAGGAATCAC-3	179
		5-ATCTTTGCACCGACTCCATC-3	
*crem*	XM_005171403.2	5-GGAACAACACCATCAGATCC-3	226
		5-CCTGAGTGATTGCAATGTACTG-3	
*ssha*	NM_131063	5-GTCAGTCTTACCTTTCGCATCC-3	1134
		5-GACCGCTATCATCAACAACCA-3	

### Reactive oxygen species (ROS), superoxide anion, nitric oxide detection, and quantification of AP sites

ROS were detected by one of several dyes obtained from Invitrogen (Eugene OR, USA). General ROS were detected by CM-H_2_DCFDA, superoxide anion was detected by MitosoxRed, and nitric oxide (NO) was detected by DAF-FM acetate. They are activated by interaction with ROS to which they are sensitive and only fluoresce upon exposure to light of the appropriate exciting wavelength. Dyes were added to fish water at a final concentration of 10 µM for CM-H_2_DCFDA and DAF-FM acetate, or 1 µM for MitosoxRed. After embryos were washed three times in fish water to remove exogenous dye, they were examined by confocal fluorescence microscopy. Eight to ten embryos were examined in each group.

To quantify AP sites, genomic DNA was prepared from Apex1 knockdown embryos and controls using QIAamp DNA Mini Kit (Qiagen, Boston, MA, USA) in the presence of deferoxamine (0.1 mM) to prevent oxidation during DNA preparation^68^. AP site assay was performed using the aldehyde reactive probe from Dojindo (Dojindo Molecular Technologies, Inc., Rockville, MD, USA) according to the manufacturer’s instructions. Two hundred embryos were used for each group.

### Hydrogen peroxide treatment and detection of oxidative damage to DNA by 8-oxo-deoxyguanosine (°G) immunostaining

For exposure to H_2_O_2_, embryos at the 64 cell stage (2 hpf) were incubated in fish water or fish water containing different concentration H_2_O_2_ for 4 h and washed three times with fish water to remove exogenous H_2_O_2_. Toxicity assayed at 6 hpf by microscopic examination indicated an LC_50_ of 0.31% peroxide. For anti-8-oxo-deoxyguanosine (°G) antibody staining, embryos were soaked in 4% paraformaldehyde (PFA)/phosphate buffered saline (PBS) overnight at room temperature and pre-chilled for 30 min in −20 °C acetone. After three washes with ×1 PBST for 5 min each in 0.5% PBS/Triton, embryos were treated with 10 µg/mL protease K for 10 min followed by post-fixation with 4% PFA/PBS for 30 min and three 30-min washes with 0.5% PBS/Triton. Subsequently, embryos were treated with RNase, incubated in 10% sheep serum for 2 h to block nonspecific staining, and then placed in 10% sheep serum containing anti-°G antibody (Trevigen, Gaithersburg, MD, USA) for 4 h. After three 30-min washes, embryos were stained with FITC-conjugated secondary antibody (1:250, Sigma Chemical Co., St. Louis MO, USA) in 10% sheep serum and examined by fluorescence microscopy using a Leica stereomicroscope (Bannockburn, IL, USA). For antibody staining, 30 embryos were used for each group.

### Brain ventricle visualization using Texas Red injection

Apex1 knockdown and control embryos were allowed to develop to 24 h post fertilization (hpf), at which time the brain ventricle was visualized by direct injection of Texas Red (5% in 0.2 mol/L KCl, Sigma Chemical Co., St. Louis, MO, USA) as described^46,59^. Fifteen embryos were used for each control or knockdown group.

### Plasmid construction and capped RNA synthesis

To construct the pApex1-eGFP plasmid, the 3.2 kb Apex1 promoter preceding the ATG start codon was cloned from zebrafish genomic DNA using Apex1 P primers (Table 1). After digestion with *XholI* and *BamHI*, the Apex1 promoter sequence was inserted into peGFP-N3 vector between the *XhoI* and *BamHI* sites to displace the original cytomegalovirus promoter^69^. Construction of pCS2+-Creb1 and pCS2+-Apex1 plasmids is described by Wang^16^ and Pei^18^. mMACHINE SP6 Kit (Applied Biosystems, Austin, TX, USA) was used to synthesize capped RNA as described^18^.

### Preparation of antisense probes and whole mount in situ hybridization

Apex1 MO knockdown embryos and controls were collected at 24 hpf. *fezf2*, *otx2*, *egr2a*, and *pax2a* were cloned from a zebrafish cDNA library with five primer pairs including SP6 promoter sequences, respectively. NCBI accession numbers and primer sequences are listed in Table 1. For generation of corresponding antisense probes, PCR products with SP6 promoter were used as templates for synthesis of DIG-labeled antisense RNA (Roche Diagnostics, Mannheim, Germany). RNA probes were purified using RNeasy columns (Qiagen, Boston, MA, USA). Whole mount in situ hybridization was performed as described^69^ with 20 embryos/group.

### Acridine orange assay

Both Apex1 MO knockdown embryos and controls at 24 hpf were incubated with 5 µg/mL acridine orange (Sigma Chemical Co.) in E3 medium (5 mM NaCl, 0.17 mM KCl, 0.33 mM CaCl_2_, and 0.33 mM MgSO_4_) at 28.5 °C for 30 min, washed with E3 medium three times, and examined with a Leica fluorescence stereomicroscope (Bannockburn, IL, USA). Fifty embryos were used for each group.

### Fluorescence immunocytochemistry

After 1-cell zebrafish embryos were micro-injected with 2 nL different concentrations MOs (control, 0.1, and 0.2 mM Apex1 MO), they were allowed to continue development for 48 h before harvest and preparation for sectioning. Brains were sliced on a cryostat in the vertical plane at a thickness of 14 μm, mounted onto coated slides, and stored at −20 °C until fluorescent immunohistochemical staining was performed^70^. Briefly, sliced brains were fixed in 4% paraformaldehyde, dehydrated in methanol, washed and incubated overnight with polyclonal rabbit antibodies directed against Apex1, Polb, or Creb1, or with polyclonal goat anti glial fibrillary acidic protein (GFAP) (Abcam, Inc., Cambridge, MA, USA) at 1/100 dilution to identify astrocytes (glial cells) and ependymal cells of the nervous system. Subsequently slides were washed and incubated with TRITC or FITC-conjugated secondary antibodies (Invitrogen, Life Technologies, CA, USA). Slides were examined by confocal microscopy (LSM 700, Carl Zeiss, Germany) for capture of fluorescence distribution.

## Supplementary information

Supplementary Figures

Supplementary figure legends
