# Supplementary figures and images for: AP endonuclease 1 (Apex1) influences brain development linking oxidative stress and DNA repair
Source: Cell Death Dis. 2019 Apr 25;10(5):348. doi: 10.1038/s41419-019-1578-1 (PMC6484078; doi:10.1038/s41419-019-1578-1)

Supplementary Figures


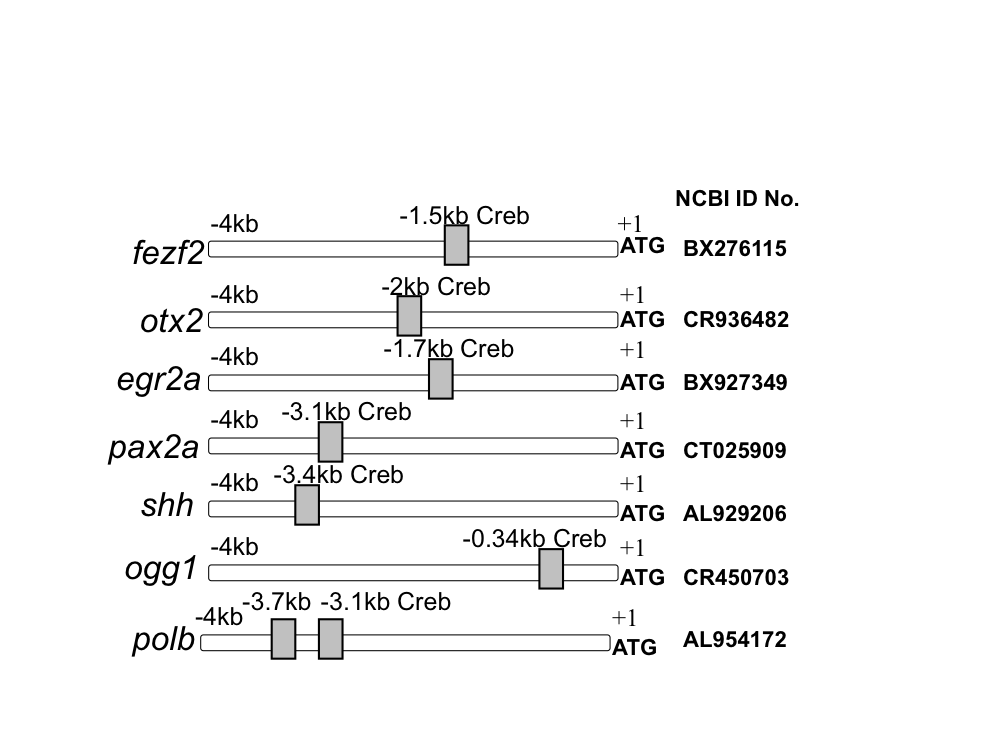


**Fig. S1.**


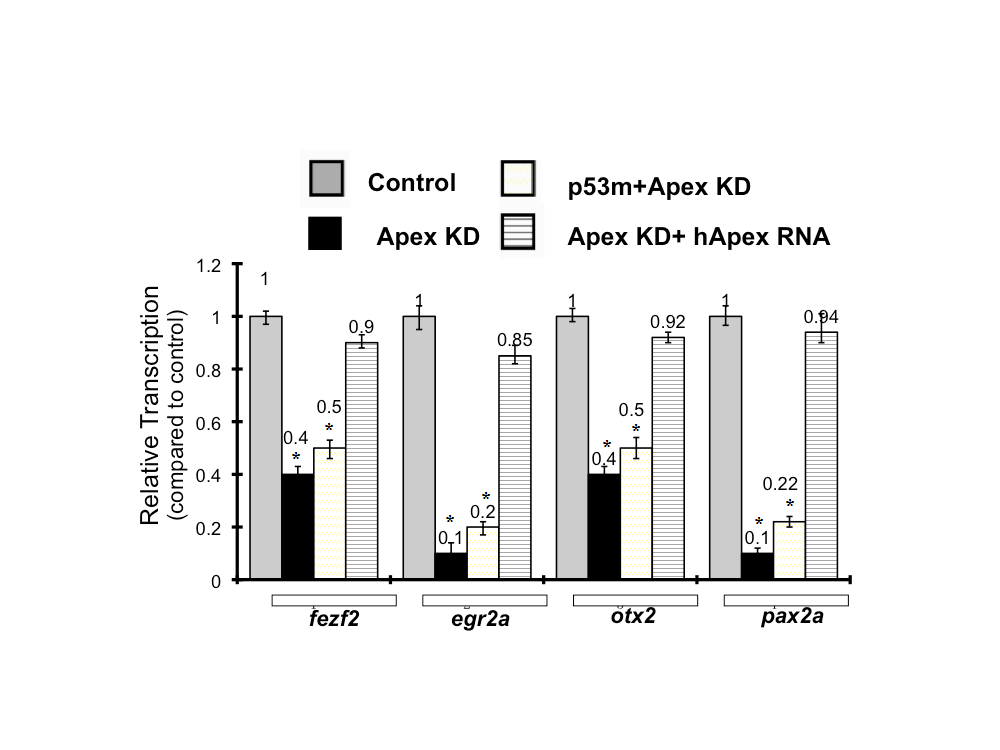


**Fig. S2.**


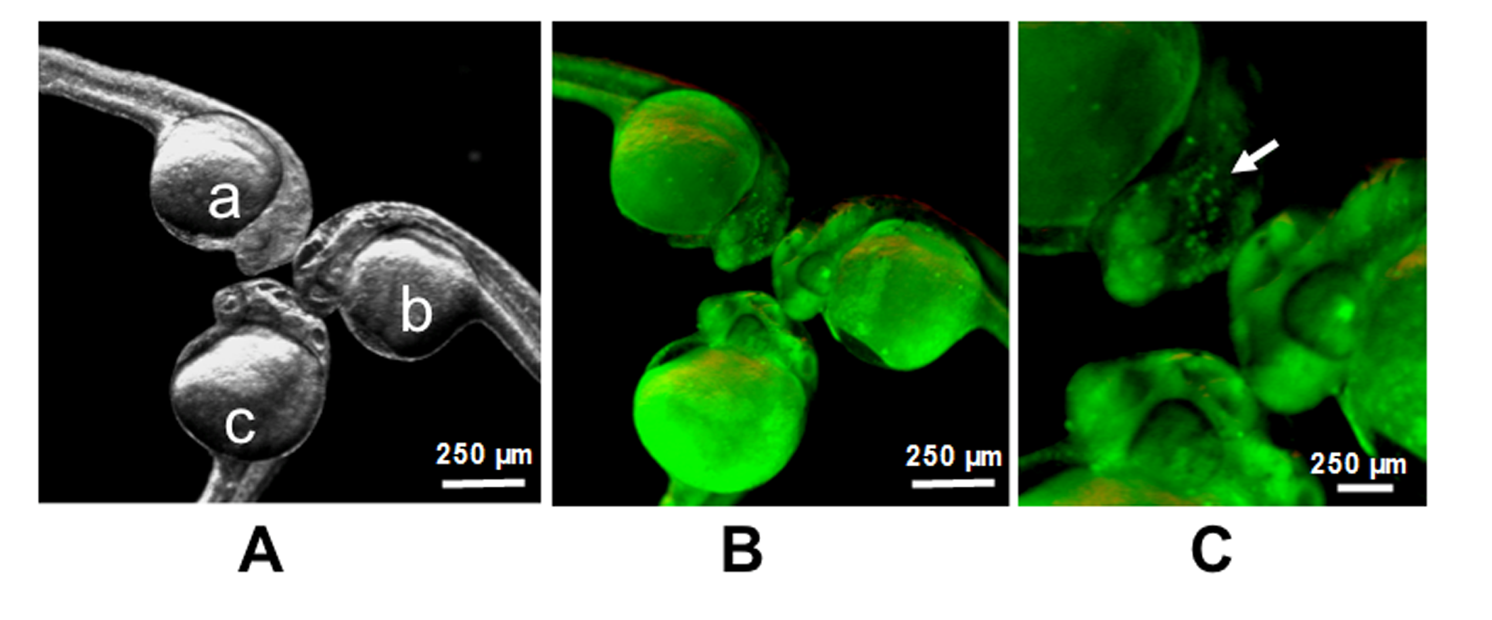


**Fig. S3.**


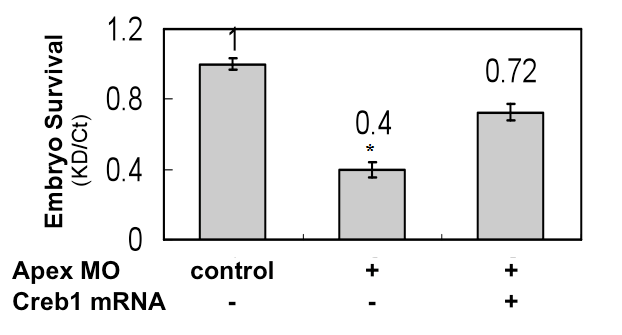


**Fig. S4.**


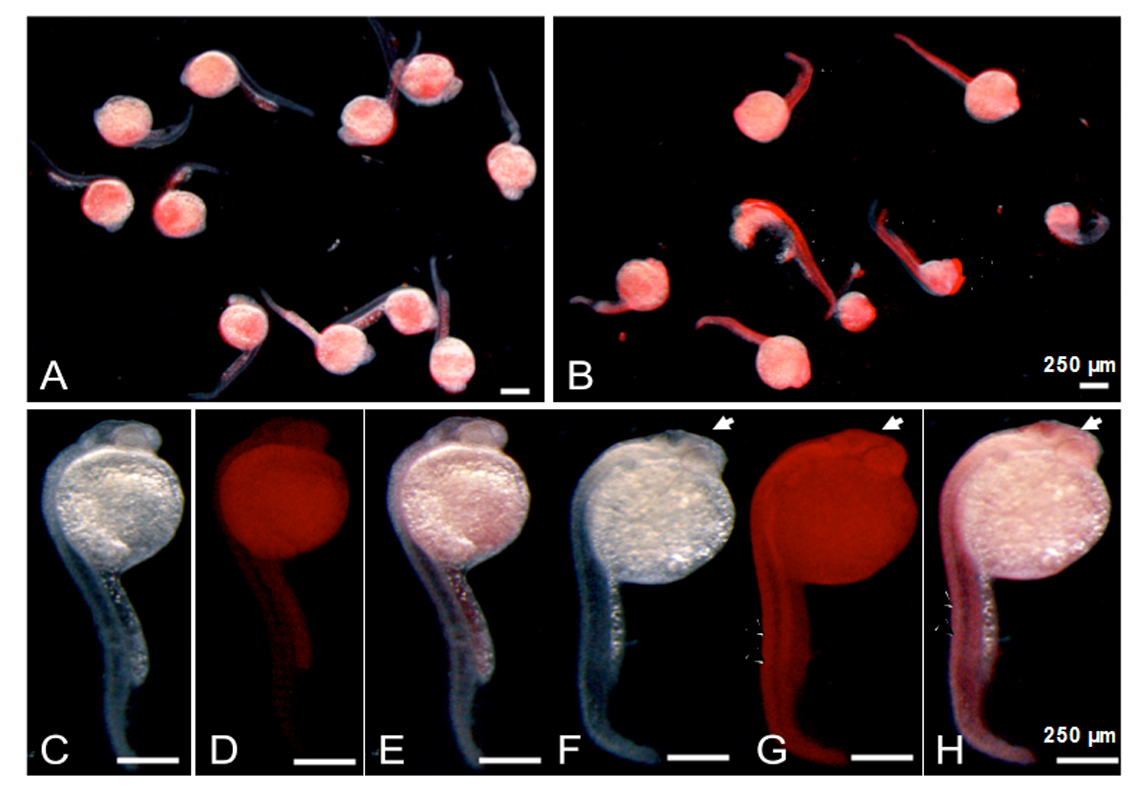


**Fig. S5.**

Supplement: Supplementary file 1 — Supplementary Figures [file 41419_2019_1578_MOESM1_ESM.docx]
