# Supplementary material for: AP endonuclease 1 (Apex1) influences brain development linking oxidative stress and DNA repair
Source: Cell Death Dis. 2019 Apr 25;10(5):348. doi: 10.1038/s41419-019-1578-1 (PMC6484078; doi:10.1038/s41419-019-1578-1)
Supplement: Supplementary file 2 — Supplementary figure legends [file 41419_2019_1578_MOESM2_ESM.docx]

**SUPPLEMENTAL FIGURE LEGENDS**

**Fig. S1. Location of Creb1 binding sites in promoters of genes examined in *in situ* studies.** Locations of Creb promoter sites in genes used here to study brain development are shown as boxes.

**Fig. S2. Transcription of four key brain transcription factors is reduced after Apex1 knockdown in wild-type and p53 mutant embryos but is rescued by co-injection of mRNA for human *apex1*.** Transcripts of *fezf2*, *egr2a*, *otx2* and *pax2a* were quantified by qRT-PCR after Apex1 knockdown in wild-type and p53 mutant embryos and after rescue by co-injection of capped mRNA for human *APEX1.* Embryos harvested at 24 hpf. ß-actin was used as the endogenous reference. These data are the average of 3 independent experiments +/- SD. Significant difference is indicated by **p* < 0.05.

**Fig. S3. Loss of Apex1 protein results in apoptosis dependent on p53.**

Wild-type embryos were microinjected with dye, 0.2 mM Apex1 MO, and 0.2 mM Apex1 plus p53 MO. After developing for 24 hpf, they were then incubated with acridine orange to detect apoptosis. **A**. Bright field image for wild type embryo with Apex1 knockdown (a), control embryo injected with vehicle (b), wild type embryo with 0.2 mM Apex1 plus p53 MO (c). **B.** Embryos shown in Panel A examined by fluorescence microscopy for apoptosis. **C**. Higher magnification of B to show apoptosis in wild type embryo with Apex1 knockdown but none in both p53 and Apex1 knockdown embryo (white arrow). The experiment was repeated 3 times and 30 embryos were used for each group.

**Fig. S4. Overexpression of Creb1 restores embryo viability after Apex1 knockdown at 24 hpf.** Embryo survival ratio was increased when injected with both Apex1 MO and Creb1 capped RNA compared with only Apex1 MO injection. Embryos were subjected to Apex1 knockdown with or without co-injected of capped *creb1* mRNA. The results are means ± SEM of three replicate samples. Significant difference is indicated by **p* < 0.05.

**Fig. S5. Increase of oxidative stress causes brain abnormalities and increased °G.**

Exposure to 1.5% hydrogen peroxide for 0.5 h at 50% epiboly results in abnormality of hindbrain ventricle height and forebrain size, and increase of oxidative DNA damage in 24 hpf embryos as detected by immunostaining with TRITC-labeled anti ^o^G mouse monoclonal antibody. Panels A, C, D, and E are control embryos, while panels B, F, G, and H are hydrogen peroxide treated embryos. Panel A and B are total views of wild type embryos and Apex1 knockdown embryos stained with TRITC-labeled anti ^o^G mouse monoclonal antibody, respectively. Panels C and F were examined in bright field microscopy; panels D and G were examined in fluorescence microscopy; panels E and H were merged with bright field and fluorescence view. Arrows indicate flattened head and enhanced accumulation of °G in treated embryo. These experiments were repeated three times with similar results.
